# News

**DOI:** 10.1155/S1463924680000156

**Published:** 1980


### Gamma-ray spectrum of natural Ti

The capture gamma-ray spectrum of natural Ti 'A note on the capture gamma-ray spectrum of natural Ti produced by thermal neutrons' is the subject of a paper by M G Sowerby of AERE Harwell. The data on the spectrum are reviewed.
*HMSO, 49 High Holborn, London WC1V 6HB, UK.*

### Sulphur analyser

A bulletin, published by Fisher Scientific details how their sulphur analyzer, by employing a specific titration reaction with microprocessor control, provides an instrumental approach to sulphur analysis. It is applicable to all metallurgical products, has a short analysis time and is essentially independent of sample characteristics. Data is provided demonstrating the linearity, accuracy and precision of results obtainable with steel samples.
*Fisher Scientific Co, 711 Forbes Ave, Pittsburgh, PA 15219, USA.*

### Nucleonics accessories brochure

Packard Instrument have published a new brochure giving details on their range of scintillation cocktails, reagents, vials and reference materials for liquid scintillations and automatic gamma counting techniques. The brochure which contains details on over 250 separate items of scintillation counting consumables should be of interest to anyone who uses radioassay techniques in the laboratory.
*Copies of the brochure are available from Packard Instrument Ltd, 13-17 Church Road, Caversham, Berks, UK.*

### Preventative maintenance schemes

Technicon's planned preventative maintenance schemes have been drawn up to provide the users of their equipment with systems which run consistently at maximum performance. Three schemes are available including an annual overhaul, check visits and emergency visits or a loan module service depending which scheme is opted for.
*A leaflet giving further details of the service schemes is available from Compagnie Technicon SA, 39, Boulevard de la Muxette, F-95140 Garages-les-Gonesse, France.*

### Clinical laboratory standards

The 1978 annual report of the National Committee for Clinical Laboratory Standards is now available. The report lists details of the committee, a financial statement and a summary of the 1978 activities. A list of the NCCLS active membership as at 31 January 1979 is given.
*NCCLS, 771 East Lancaster Ave, Villanova, PA 19805, USA.*

### Switches booklet

A leaflet from Techmation describes the Giannini range of Enviro-Switches. The contacts of the switches are enclosed within a welded chamber and are operated externally by a magnetic action. The leaflet describes the available switch materials and such configuration as push-button switches, rotary switches, plungers, key-lock switches and limit switches.
*Copies of the leaflet 'Do your switches switch off when the going gets tough?' are available from Techmation Ltd, 58 Edgware Way, Edgware, Middx, UK.*

### Automation of chemical oxygen demand analyses

A paper is available from Techmation which describes the automatic measurement of chemical oxygen demand by Johnson COD meter in the sewage systems of a Swedish pulp mill. Advantages of the COD meter, including the automation of laboratory work and high measuring capacity are discussed together with the results of correlation studies between BOD and COD analyses for various wastewater types.
*Techmation Ltd, 58 Edgware Way, Edgware, Middx, UK.*

### Printed circuit board components

A free catalogue has been published by Sealectro covering their ranges of pins, jacks and links for printed circuit boards, multi-layer and flex circuits. Intended as a guide to the readily available styles from their existing pin jacks and terminal pin ranges, this catalogue also includes a new range of barb and swage mounting pins.
*Sealectro Ltd, Walton Road, Farlington, Portsmouth Hants, UK.*

### Chromatography newsletter

The August 1979 issue in the continuing series of Chromatography Newsletters is available from Perkin-Elmer. Articles on advanced technology applications in both liquid and gas chromatography are featured in the Newsletter, seven of which in this issue are written by outside laboratories.
*Copies of the newsletter can be obtained from Perkin-Elmer Ltd, Post Office Lane, Beaconsfield, Bucks, UK.*

### News

### National Committee for Clinical Laboratory Standards (NCCLS)

The National Committee for Clinical Laboratory Standards is an American (United States of) organisation that develops and publishes standards for clinical laboratories. By the voluntary implementation of these standards in United States laboratories, it is hoped that the imposition of regulations by outside agencies will be avoided.

The active membership of the committee comes from organisations covering the widest range of interests. The organisations include Industry, Government, Trade Associations, as well as Professional and Learned Societies. The corresponding membership includes hospitals, clinics and medical schools throughout the United States. It is by such wide consultation that consensus standards can be achieved.

The documents published cover a comprehensive range of laboratory problems. These include consideration of hardware requirements, instrument servicing, materials, as well as standards for the evaluation of experiments, calculation of results and the expression of data.

The standards are of three types, Approved, Tentative, and Proposed. Comments are invited from any interested reader and not only from those active in the NCCLS. It is evident that efforts are made to keep the standards as up to date and as relevant as can reasonably be achieved. As well as the revision and republication of existing standards, approximately twenty new standards are to be published in the near future.

All the standards are produced to the same high quality. The documents are set out clearly and the subsections are clearly referenced. Also where relevant, ample bibliography is given. Although intended for the clinical chemist the standards can yield useful data to those with an interest in related fields.

The following documents are pertinent to Automatic Analysis:

PSC-7 Guidelines for Kinetic Analysis of Enzyme Reactions.

PSEP-2 Protocol for Establishing Performance Claims for Clinical Methods – Introduction and Performance – Check experiment.

PSEP-3 ibid – Replication Experiment.

PSEP-4 ibid – Comparison of Methods Experiment.

PSC-12 Definitions of Quantities and Conventions related to Blood pH and Gas Analysis.

TSC-5 Methodological Principles for establishing Principal assigned values to Calibrators.

### Swansea summer school of automatic chemical analysis

July 6–11 1980 University College Swansea.

An intensive short course covering all aspects of automatic analysis will be held in Swansea. The programme will comprise of a series of authoritative lectures given by a team of world experts in automatic chemical analysis, tutorial sessions and practical hands on experience of some of the latest automatic instruments. In addition students will consider the automation strategy necessary to solve a real world course problem. The following experts in automation have already indicated their attendance, Professor H.L. Pardue, Professor M. Bonner Denton, Professor J. Ruzicka, Dr. J. Betteridge, Dr. F.L. Mitchell, Dr. P. Saunders, Dr. D. Deans, Dr. P.B. Stockwell, D.G. Porter and J.K. Foreman. For further details of this course suitable for both practising chemists and managers alike please write to Dr. J. Betteridge, University College of Swansea. Course fee is £270 – early booking necessary due to limited accommodation.

### Programming microprocessors for industrial measurement and control

A two-day seminar on Programming microprocessors for industrial measurement and control is being jointly organised by Sira and Warren Spring Laboratory. It will be held on 29-30 April 1980 in London.

The aims of the seminar are to clarify the various classes of need for programming, in relation to the several levels of programmability that should be provided by the new generation of microprocessor-based products, to present the views of the protagonists of assembly level and of high level languages, and to draw some conclusions regarding the best approach to adopt in programming microprocessors at each of the three stages: product development, installation and in-use operation. The programme comprises eight sessions including software and programming techniques, device programming, system programming and validity and testing of software.

*Enquiries should be sent to Mrs R G Keiller, Sira Institute Ltd, South Hill, Chislehurst, Kent BR7 5EH, UK.*

### Product News

### Capillary dispenser

The Camag capillary dispenser system offers the high precision of sample positioning that is required for automatic chromatogram scanning.

It is loaded with a disposable capillary by pushing the pipette holder into the mouth of the dispenser. Once filled with sample solution the holder is placed in the magnet head of their nanomat from where it is dropped on to the layer surface at an adjusted speed and for a regulated contact time. With the automatic repetition device of the nanomat the capillary content can be delivered in small increments. Pipette holders and capillaries in dispensing magazines are available in sizes ranging from 0.5 - 5$\mu$l.

*Camag, Homburgerstrasse 24, CH-4132 Muttenz, Switzerland.*

### Cassette data recorder

A compact, portable and weatherproof recorder, the CR50, which records on standard cassettes has been introduced by Grant Instruments. The recorder has been used to monitor conditions in the transportation of refrigerated food and as a remote unattended weather recorder. Inputs can be in the form of resistance, voltage, pulse counting or digital information. Sensors which can be used with the recorder include thermistors, platinum resistances, thermocouples, wet and dry psychrometers and relative humidity sensors. The recorder is designed to work in a range of temperature from -20 to +60°C. Standard type cassettes are used, together with easily available standard batteries.

On every cycle a recording is made from each sensor in turn, followed by a recording of real-time and an identification number. Cycles are initiated at intervals which can be pre-set by the user between 1 and 999 minutes on a 3-digit thumbwheel switch. Completion of a recording cycle takes under 2 seconds.

*Grant Instruments (Cambridge) Ltd, Barrington, Cambridge CB2 5QZ, UK.*

### Radiation contamination monitor

Laboratory Impex have recently introduced a new high sensitivity beta and gamma contamination monitor. The instrument, the LB1210B, offers a detection limit of $10^{-5}$ uCi/cm$^2$. The 100 cm$^2$ xenon filled detector fitted to the monitor is designed to survey either rough or smooth surfaces and can be adapted for use as an exit or atmosphere monitor without loss of sensitivity due to temperature or humidity fluctuations. The detector can be operated single-handed. Radiation levels are indicated on a large meter scale with x 1 or x 10 time constant readout modes. The x 10 mode operates a 20 second integrate cycle giving high measurement precision. A built in audible alarm with an adjustable threshold indicated the presence of a contaminated area and low battery power is indicated by an LED warning light. The unit can be operated direct from a 220/240 volt mains supply.

*Laboratory Impex Ltd, Lion Road, Twickenham, Middx, UK.*

### Trace element analysis

The plasma emission spectrometer Spectraspan III from Techmation performs simultaneous multi-element analysis to determine trace elements in stainless steels. Sample preparation for most analyses consists of dissolution in the minimum amount of 1:1 HCl/ $HNO_3$ necessary and dilution to a concentration of 2% is sufficient. An echelle grating spectrometer with a resolution claimed to be 10x better than other atomic analysis instruments is incorporated and it permits the use of previously avoided spectral lines such as the niobium 309.4 nm line which has closely associated water bands. The instrument has many applications in the analysis of wear metals in heavy engineering and aircraft industries.

*Techmation, Ltd, 58 Edgware Way, Edgware, Middx, UK.*

